# Early CER Strategies to Recruit Black 80+ year olds in the SuperAging Research Initiative

**DOI:** 10.1002/alz.091560

**Published:** 2025-01-09

**Authors:** Phyllis Timpo, Amanda Cook Maher, Chandler Zolliecoffer, Annalise Rahman‐Filipiak, Gabrielle A Lincoln, Sophia Moore, Avemaria Awosika, Rebecca Devine, Hannah Peirce, Janessa Engelmeyer, Darby J. Morhardt, Ozioma C Okonkwo, Monica Bruce, Sarah R Lose, Felicia Goldstein, Antoine R Trammell, Mary Locke, Monica W Parker, Ivan Culum, Elizabeth Finger, Angela C Roberts, Emily J Rogalski

**Affiliations:** ^1^ University of Chicago, Chicago, IL USA; ^2^ University of Michigan, Ann Arbor, MI USA; ^3^ Northwestern University Feinberg School of Medicine, Chicago, IL USA; ^4^ University of Wisconsin‐Madison School of Medicine and Public Health, Madison, WI USA; ^5^ University of Wisconsin ‐ Madison, Madison, WI USA; ^6^ Wisconsin Alzheimer’s Disease Research Center, School of Medicine and Public Health, University of Wisconsin‐Madison, Madison, WI USA; ^7^ Emory University School of Medicine, Atlanta, GA USA; ^8^ Emory University Goizueta Alzheimer’s Disease Research Center, Atlanta, GA USA; ^9^ Western University, London, ON Canada; ^10^ University of Western Ontario, London, ON Canada; ^11^ Healthy Aging & Alzheimer’s Research Care (HAARC) Center, Healthy Aging & Alzheimer’s Research Care (HAARC) Center, The University of Chicago, Chicago, IL USA

## Abstract

**Background:**

Community engaged research (CER) has been critical for increasing diversity in Alzheimer’s studies. However, the effectiveness of CER for engaging a diverse population of ‘SuperAgers,’ has been largely unstudied. SuperAgers are individuals age 80+ with superior episodic memory. The multi‐site SuperAging Research Initiative (SRI) seeks to recruit at least 500 SuperAgers and similarly aged controls, including 40% who identify as Black. This report summarizes initial CER strategies, implementation of new strategies, and enrollment success across the first ∼18 months.

**Method:**

Semi‐structured interviews were conducted at each site to assess current CER strategies and perceptions of facilitators/barriers to CER approaches. Interviews were transcribed and concept mapping was used to identify key themes across sites. Key themes are reported here along with recruitment outcomes across the sites over the first ∼18 months. Initial responsive actions are described.

**Result:**

SRI sites were selected based on their historical success engaging and recruiting Black older adults in cognitive aging research; however, identification and recruitment of SuperAgers was new for most sites. Results revealed strong current community engagement practices across sites. Emergent themes included prioritizing diverse staff, barriers to CER, strengths of the multi‐site design, the value of research to participants. Sites also implemented several CER strategies including building/enhancing community partnerships, providing community presentations, and engaging community members and enrolled participants in feedback sessions related to recruitment methods. Responsive actions of the SRI included development of participant informed culturally tailored recruitment flyers, videos, and photos, which incorporated site‐level feedback. Holiday‐themed appreciation gifts were provided to enrolled participants.

Initial enrollment included 173 participants (mean age: 86.6 years). Diverse recruitment across sites ranged from 0‐27%. Diverse recruitment increased over the second half of the year, potentially suggesting early success with CER strategies.

**Conclusion:**

Initial recruitment reflects a starting point for the enrollment of Black SuperAgers and Controls. Future plans involve implementing additional study‐wide CER recruitment methods, utilizing online registries as a recruitment source, and establishing ambassador boards to boost SRI enrollment of older Black adults. Strategy success will be carefully quantified, and outcomes will be shared for future research use.

